# Patterns of Childhood Trauma and Psychological Distress among Injecting Heroin Users in China

**DOI:** 10.1371/journal.pone.0015882

**Published:** 2010-12-29

**Authors:** Zhen Wang, Jiang Du, Haiming Sun, Helen Wu, Zeping Xiao, Min Zhao

**Affiliations:** 1 Shanghai Mental Health Center, Shanghai Jiao Tong University School of Medicine, Shanghai, China; 2 Department of Obstetrics & Gynecology, University of Texas Medical Branch, Galveston, Texas, United States of America; Chiba University Center for Forensic Mental Health, Japan

## Abstract

**Background:**

Childhood trauma has been reported as a possible cause of future substance abuse in some countries. This study reports the prevalence of childhood trauma and examines its association with psychological distress among injecting drug users from mainland China.

**Methodology:**

The study was conducted in three government-operated drug rehabilitation facilities in Shanghai, China in 2007. The Early Trauma Inventory Self Report-Short Form (ETISR-SF) was used to evaluate 4 types (general, emotional, physical and sexual) and severity of childhood trauma, and the Symptom Checklist-90-Revised (SCL-90-R) to evaluate psychological distress.

**Principal Findings:**

Among 341 injecting drug users who completed the study, about 80% reported one or more types of childhood trauma, specifically 53% general trauma, 56% physical abuse, 36% emotional abuse and 26% sexual abuse. Compared to female injecting drug users, males reported significantly higher scores of general trauma and physical abuse, but lower sexual abuse scores. Hierarchical linear regression analyses showed that greater physical and emotional abuse in childhood predict greater current psychopathological distress among these injecting drug users in China.

**Conclusions:**

The results reveal a high prevalence of childhood trauma among injecting drug users in China, and it is comparable to other similar studies in Western countries. It is important to consider the role of childhood trauma in the prevention and treatment of substance abuse.

## Introduction

Childhood trauma, including physical abuse, sexual abuse, and emotional abuse/neglect in childhood, is a significant risk factor for many psychiatric disorders later in life, such as substance abuse, borderline personality disorder, depression, and posttraumatic stress disorder [1], [2], [3], [4], [5], [6]. Rates of childhood trauma in the general population of western countries varied from about 20% to over 40% in different studies and in different countries [7], [8], [9], [10]. In China, the only available study showed much lower rates of childhood trauma: 0.1% reported childhood sexual and physical abuse among a group of Chinese factory workers, and 7.8% reported childhood abuse among schizophrenia inpatients in Shanghai, China [11]. The results may be due to the instrument used in the sutdy— Dissociative Disorders Interview Schedule [12]— which is not specifically designed to measure childhood trauma. Such a low estimated rate of childhood trauma in Shanghai warrants further investigation.

Studies in the United States found that childhood trauma is related to future substance abuse [13], [14], [15]. A much higher prevalence of childhood trauma among cocaine, heroin, and alcohol abusers, ranging from 60% to 90% [14], [15], [16], [17], [18] has been reported. Since 1990, drug abuse in China has been worsening—with a 20-fold increase from 70,000 official registered drug abusers in 1990 to 1.16 million in 2005. In 2005, the number of all drug users was estimated to reach 3.5 million in China [19], [20]. Furthermore, compared with 1991, more than 4 times as many drug-related offenses were recorded in 2005 [21]. Thus, to better understand how childhood trauma may impact drug abuse later in life and to provide adequate intervention strategies in the fast growing drug abuse populations, including heroin users in China, an accurate report of childhood trauma in such populations is much needed. As mentioned above, traumatic experience in childhood could also lead to other psychiatric conditions, psychological symptoms or disorders later in life [1], [2], [3], [4], [5], [6]. However, among Chinese drug abusers, the relationship between childhood trauma and psychiatric disorders has never been explored. This study aims to find the prevalence of childhood trauma and to examine its potential association with psychological symptoms among a substance abusing population in China. We hypothesized that childhood trauma is a common phenomenon and associated with psychological distress in injecting drug users in China.

## Methods

### Ethics Statement

This research protocol was approved by the Ethics Committee of the Shanghai Mental Health Center and written informed consent was obtained from all study participants.

### Participants and setting

This study was conducted as the baseline part of a 5-year longitudinal epidemiological study of injecting drug users (IDUs) in Shanghai. The main aim of the five year study is to estimate gender differences in the prevalence of HIV risk behaviors and the prevalence of HIV, HCV and HBV among IDUs. The study design uses convenience sampling to recruit any patients who meet study inclusion criteria and who were mandated to undergo drug rehabilitation programs. Once consented, the study participants will be followed for 5 years. The study samples were recruited from April to September 2007 according to the following inclusion criteria: (a) meeting the DSM-IV diagnostic criteria for substance dependence as assessed by the Structured Clinical Interview for DSM-IV Axis I Disorders (SCID-I) [22]; (b) being at least 18 years of age; (c) having injected drugs during the 30 days prior to admission to a rehabilitation program; (d) having no severe physical conditions (such as an acute stage of cardiovascular disease, cerebrovascular disease, or neurodegenerative diseases), and (e) having the cognitive capacity to give consent.

At each study site, potential participants were identified from a computerized database of heroin addicts. Individuals over 18 years of age, who were identified at intake assessment as DSM-IV heroin dependent, were approached and consented by trained physician interviewers from Shanghai Mental Health Center, Shanghai Jiao Tong University School of Medicine. Since this study has been funded by the National Institute of Health in the United States, the study is also compliant with their human subject protection requirements.

After the study procedures were explained, each potential participant reviewed a consent form and then was asked to repeat the main points to check for understanding. If he or she did not clearly understand, interviewers explained again. If the potential participant still did not understand, he or she was not enrolled. At baseline, a structured interview was conducted in person by trained physicians. The interviewers collected each participant's information, such as demographic characteristics and drug use history (such as onset age of drug use and the duration of drug use), and then administered an additional questionnaire designed for this study. After the face-to-face interview, participants were asked to complete a set of self-administered questionnaires including ETISR-SF and The Symptom Checklist-90-Revised (SCL-90-R). Herein, the cross-sectional data at baseline of the parent longitudinal study were used for statistical analyses. The response rate of this baseline study is 100%, which is usually expected in China.

### Measures

Childhood trauma was assessed using ETISR-SF [23]. This instrument contains a total of 27 items, including 11 items for general trauma, 5 items for emotional abuse, and 6 items for sexual abuse. Participants responded “yes” ( = 1) or “no” ( = 0) to each item. Within each of these 4 domains, an index was created by counting all responses, and a total score was a summation of all items. Higher scores in each domain indicate more traumatic experiences. The ETISF-SF has an acceptable reliability, indicated by Cronbach's alphas for these 4 domains ranging from 0.70 to 0.87 [23]. The Chinese version of ETISR-SF, which was tested among a group of depression patients and drug user shows a similar range of Cronbach's alpha values from 0.67 to 0.84 [24]. In the current study, the reliability of ETISR-SF ranged from 0.66 to 0.82.

Psychological distress was assessed by using the SCL-90-R [25], a 90-item self-report measure of global psychopathological symptoms. The Chinese version of the SCL-90 has a good reliability, indicated by a Cronbach's alpha of 0.97 [26]. Respondents rated items on a five-point scale reflecting their distress during the past 7 days. Scores were generated for nine symptom scales and an overall Global Severity Index (GSI). Because several SCL-90 subscale scores were highly correlated, we used only the GSI score, which has been widely considered the single best global measure of psychological distress.

Covariates include demographic characteristics such as gender, age, marital status, education, and employment status, and drug use history, such as onset age of drug use and the duration of drug use. Then, recent drug use was verified by urinalysis results from each participant's admission record. If self-reported drug use information did not match the urinalysis result, a positive urinalysis results would be used for the study.

### Statistical Analysis

All data was entered twice and cleaned by checking for expected ranges, presence of abnormal values, and whether the distribution of variables met assumptions of statistical tests. Bootstrap *t*-tests were used for the comparison because childhood trauma data had an abnormal distribution. Bootstrap analysis was performed using STATA 10.0 (STATA Corporation, College Station, TX).

Then SPSS 13.0 for Windows (SPSS Inc. Chicago, IL) was used for all remaining statistical analyses. Pearson correlation analyses were used to investigate associations between childhood trauma and psychological distress. Further, in order to test the contribution of each domain of childhood trauma to psychological distress, a hierarchical linear regression analysis was conducted in a sequence of two models. In each model, psychological distress, measured by the SCL-90 GSI score, was the dependent variable. Model 1 accounted for the effects of covariates, age, gender, education, marital status, employment, and duration of drug use were entered into the regression equation. Four types of childhood trauma were entered in Model 2. Statistical significance at *P*-values less than 0.05 was reported for all analyses.

## Results

### Participant characteristics

All 341 participants were diagnosed with DSM-IV heroin dependence. The reported average daily heroin dose of the participants prior to being admitted to rehabilitation programs was 0.98 g. Among the participants, 30.3% reported using methamphetamine, 39.1% using alcohol, 16.5% using marijuana and 11.5% using other illegal drugs during their life time. Table 1 presents the demographic and clinical characteristics of the participants. No significant gender differences were found by demographic factors except marital status.

**Table 1:** Participant characteristics of injecting drug users in China (n = 341).

	Total (n = 341)	Male (n = 123)	Female (n = 218)	*t*
	Mean (SD)	Mean (SD)	Mean (SD)	
Age (in years)	31.2 (6.1)	31.1 (6.2)	31.3 (6.0)	0.26
Years of education	10.4 (1.8)	10.2 (1.9)	10.5 (1.8)	1.09
Onset age of drug use	21.7 (7.4)	21.3 (4.3)	21.8 (8.6)	0.58
Drug use duration (in years)	9.6 (7.9)	9.7 (5.0)	9.5 (9.1)	0.34

	n (%)	n (%)	n (%)	*x^2^*
Unmarried	186 (54.5)	83 (66.7)	104 (47.7)	11.4 a
Unemployed	258 (75.7)	91 (74.0)	167 (76.6)	0.29

a *P*<.01.

### Childhood trauma

About 80% of the participants reported at least one type of childhood trauma. The rates of each type of childhood trauma are presented in Table 2. Compared with females, males reported experiencing significantly more severe general trauma and physical abuse, but less sexual abuse. Approximately 20.0% of males and 29.4% of females had been sexually abused, including molestation, and 2.4% of males and 8.3% of females experienced forced sexual penetration.

**Table 2:** Comparison of childhood trauma by gender among injecting drug users in China.

	Total (n = 341)		Male (n = 123)Mean(SD)	Female (n = 218)Mean(SD)	*z*	*OR (95% CI)*	*Adjusted OR (95% CI)* a
	N(%)	Mean(SD)					
General Trauma	180 (52.8)	1.01 (1.23)	1.26(1.42)	0.86(1.09)	2.68 b	1.23 (1.00–1.51)	1.27 (1.02–1.59)
Physical Abuse	191 (56.0)	1.18 (1.33)	1.80(1.44)	0.83(1.11)	6.03 b	1.92 (1.56–2.37)	1.95 (1.57–2.43
Emotional Abuse	122 (35.8)	0.64 (1.04)	0.68(1.00)	0.61(1.06)	0.59	1.21 (0.94–1.58)	1.26 (0.96–1.66)
Sexual Abuse	88 (25.8)	0.43 (0.85)	0.28(0.66)	0.51(0.93)	−2.66 b	1.83 (1.31–2.61)	1.94 (1.36–2.78)

a Adjusted for age, education, duration of drug abuse, marriage status, and employment status.

b *P*<.01.

### Correlations between childhood trauma and psychological distress

The mean SCL-90-R GSI score in this population was 0.7. All four types of childhood trauma measured by ETISR-SF subscales were significantly correlated with psychological distress as measured by SCL-90-R GSI scores (Table 3). The correlation coefficients between all types of childhood trauma, which are all under 0.35, are statistically significant (*P*<0.01).

**Table 3:** Correlations of four types of childhood trauma with psychological distress (GSI) (n = 341).

Variables	Global Severity Index (GSI)	General Trauma	Physical Abuse	Emotional Abuse
General Trauma	0.155	--		
Physical Abuse	0.249	0.302	--	
Emotional Abuse	0.230	0.291	0.332	--
Sexual Abuse	0.157	0.194	0.165	0.193

*P*-values for all Pearson correlation coefficients <.01.

### Hierarchical Linear Regression Analyses

From hierarchical linear regression analyses, six covariates, including age, gender, marital status, education, employment status, and duration of drug use were regressed against GSI as a first step in analysis (Model 1, Table 4). This model accounted for 0.06% of the variance in higher levels of current psychological distress (GSI). None of these variables are significantly related to psychological distress.

**Table 4:** Hierarchical regression models to assess the associations between childhood trauma and psychological distress among injecting drug users in China (n = 341).

	Global severity index of psychological distress	
	*Model 1* *(β)*	*Model 2* *(β)*
**Covariates**		
Female vs. male	0.032	0.110
Age	−0.037	0.011
Years of education	−0.028	−0.039
Married vs. not married	−0.005	0.029
Employed vs. unemployed	−0.023	−0.011
Duration of drug use	0.067	0.072
Model summary statistics	***R*** **^2^** = 0.006	
	***ΔR*** **^2^** = 0.006	
	***ΔF*** = 0.280	
**Childhood Trauma**		
General Trauma		0.066
Physical Abuse		0.227 a
Emotional Abuse		0.125 b
Sexual Abuse		0.081
Model summary statistics		***R*** **^2^** = 0.115
		***ΔR*** **^2^** = 0.109
		***ΔF*** = 3.703 c

a *P*<.01.

b *P*<.05.

c *P*<.001.

In Model 2, four childhood trauma variables were added to Model 1. Model 2 accounted for an additional 10.9% of the variance. After controlling for covariates, greater physical abuse and emotional abuse were significantly correlated with higher levels of current psychological distress.

## Discussion

This cross-sectional study reported a much higher prevalence of childhood trauma (80%) among injecting drug users when compared to the rates previously reported in the general population or other subpopulations in China. Furthermore, greater physical and emotional abuse in childhood was associated with greater psychological distress later in life in this sample.

In China, childhood trauma has been treatedas an uncommon phenomenon historically. A previous study suggested that the rates of physical and sexual abuse were low among the general population and schizophrenia patients [11]. However, in the current study, injecting drug users reported a substantially high rate 80% of participants reported experiencing childhood trauma. This rate is comparable to their counterparts' reports in Western countries [14], [15], [18], [27]. Because childhood trauma is a preventable phenomenon that occurs early in life and affects psychological functioning well into adulthood, our findings highlight the possibility of child abuse prevention as a viable means of drug abuse prevention in China.

Considering the extremely low prevalence of childhood trauma reported among the general population of China, individuals who experienced childhood trauma may tend to be more vulnerable to substance abuse. One explanation is that poor coping strategies, such as avoidance, have been demonstrated to exacerbate the impact of childhood trauma on later substance abuse [28], [29]. In general, Chinese tend to use acceptance and avoidance as ways to cope with difficult situations [30]. One survey among 512 Chinese heroin users found that over seventy percent of these patients have avoidant personality traits [31]. So, avoidance coping may be even more salient among this underprivileged subpopulation of injecting drug users who were abused during childhood. However, further investigation is warranted to determine how different coping styles may affect Chinese populations, including drug users dealing with traumatic situations. Furthermore, there are many other factors contributing to both injection drug use and childhood trauma, but the current study cannot delineate these factors.

Gender differences in childhood trauma were found in this substance abusing population. Our finding of males experiencing significantly more physical abuse than females is inconsistent with what has been reported in previous studies [14], [15], [17]. The reason for this inconsistence is not clear because few studies on childhood trauma have been done in China. One potential explanation may be cultural differences, but further study is needed. However, our study found that gender differences in sexual abuse were similar to those found in Western studies, but the rates of reported sexual abuse were much lower compared to other countries [16], [32].

Childhood trauma is known to be linked to a wide range of negative outcomes in adulthood [18], [27], [33], [34], [35], [36], [37]. In particular, levels of cumulative trauma have been associated with psychological distress [17]. Further, childhood trauma, including all types of trauma, may serve as a common etiological factor in substance abuse and psychological distress [29]. Our study showed that rather than sexual abuse, physical abuse and emotional abuse, in childhood are significantly associated with psychological distress among a drug using population in China. Braver's (1992) study in a university counseling center found that physical and emotional abuse had a greater impact than sexual abuse on subsequent adjustment. This model of childhood trauma's impact on human psychology is in line with our findings. These findings are relevant to clinical practice because sexual abuse has often been thought to be the most important type of childhood trauma.

Furthermore, stress, including childhood trauma, can increase an individual's vulnerability to addiction and relapse [38], [39]. For example, childhood abuse survivors did not benefit from treatments as much as their non-abused peers in psychological functioning or substance abuse [40]. In addition, identification of types of childhood trauma and recognition of gender differences in trauma are important to treatment outcomes. A recent study of a cocaine-dependent population found that greater severity of childhood emotional abuse was associated with an increased risk of relapse in females but not in males [41]. Considering evident gender differences in types of childhood trauma in our study, future studies are necessary to explore the interaction of different childhood traumas, gender, and treatment responses in substance abuse patients in China.

There are several limitations in this study. First, the validity of adult retrospective self-report measures of childhood trauma may be compromised by fallibility of memory and social desirability bias [29], even though it has been demonstrated to be a worthwhile method [42]. Secondly, because all participantswere abstinent from substance use for almost one year since they had stayed in the rehabilitation units for one year when they were recruited, their psychological distress levels may be lower than those in active drug users. Thus, our findings may not be generalizable to other substance using populations in China. Third, although we found a very high rate of childhood trauma among heroin users, we cannot determine if heroin addiction was indirectly related to childhood trauma or if the addiction was a result of childhood trauma because of the cross-sectional nature of this study. Fourth, since the majority of participants who show more sexual abuse, but less general trauma and physical abuse in this study were female, the sexual abuse rate might be overestimated, where as general trauma and physical abuse rates might be underestimated. Also the reasons for gender differences are more complex than what our results showed here.

In spite of these limitations, our findings provide the first estimates of the prevalence of childhood trauma in injecting drug users and demonstrate association between childhood trauma and current psychopathological distress among drug users in China. In summary, this study highlights the importance of identifying childhood trauma in drug abuse prevention and treatment in China.
